# Olfactory Memory in Depression: State and Trait Differences between Bipolar and Unipolar Disorders

**DOI:** 10.3390/brainsci10030189

**Published:** 2020-03-24

**Authors:** François Kazour, Sami Richa, Chantale Abi Char, Boriana Atanasova, Wissam El-Hage

**Affiliations:** 1 UMR 1253, iBrain, Université de Tours, Inserm, 37044 Tours, France; boriana.atanasova@univ-tours.fr (B.A.); wissam.elhage@univ-tours.fr (W.E.-H.); 2 Psychiatric Hospital of the Cross, Jal Eddib 60096, Lebanon; chantaleabicharr@hotmail.com; 3 Department of Psychiatry, Faculty of Medicine, Saint-Joseph University, Beirut 166830, Lebanon; richasami@hotmail.com; 4 CHRU de Tours, Clinique Psychiatrique Universitaire, 37044 Tours, France

**Keywords:** unipolar, bipolar, depression, olfaction, marker, recognition memory

## Abstract

Background: Changes in olfactory recognition memory may constitute sensory markers in depression. Significant differences may exist between unipolar and bipolar depression. Our study compares olfactory memory between control, unipolar, and bipolar patients in depressed and euthymic states in order to identify potential markers of depression. Methods: 176 participants were recruited in 5 groups: depressed bipolar (DB), euthymic bipolar (EB), depressed unipolar (DU), euthymic unipolar (EU), and controls (HC). The participants had a standardized clinical and olfactory assessment (olfactory memory, evaluation of pleasantness, intensity, familiarity, and emotional aspect of smells). Results: DU, DB, and EU patients had a deficit in olfactory memory compared to HC. DB patients had lower capacity to recognize new odors. DB and DU patients had more limited detection of unfamiliar odors than HC. DB patients rated odors as less pleasant compared to the other groups. All groups had lower hedonic ratings than HC. DB patients had lower emotional ratings than EU patients. Conclusions: Olfactory memory is impaired in depressive states, thus constituting a state marker of depression. Impairments in olfactory memory persist after remission of bipolar depression, thus constituting a possible trait marker of bipolarity. Hedonic rating differentiates unipolar from bipolar depression. This is the first study that identifies a sensory marker differentiating between unipolar and bipolar depression.

## 1. Introduction

Memory impairment is a frequent symptom of depression, although it is not a cardinal criterion for diagnosis [1]. According to Lam et al. (2012) [2], 39% of patients suffering from Major Depressive Disorder (MDD) present memory problems that affect their daily functioning [2]. However, it is not clear if memory symptoms persist or disappear after symptomatic remission of depression. According to DSM-5 (Diagnostic and Statistical Manual for Mental Disorders—5th edition), in successfully treated depressed patients, memory symptoms may fully abate after remission [1]. Subjects suffering from depression present memory biases in the form of preferential recall of negative information [3]. Liu et al. have also shown that depressed patients have a lower memory performance in recall measures, as well as a memory bias toward negative words [4]. Studies showed that cognitive memory biases seen in depression improve after remission [5], but formally depressed patients keep on having a greater recall of negative information when compared to never-depressed patients [6].

A depressive episode can be a presentation of either MDD (Unipolar Depression) or Bipolar Disorder (BPD) [1]. BPD differs from MDD in the occurrence of (hypo)manic episodes. Treatments of unipolar and bipolar depressive episodes vary considerably and will affect the patient’s prognosis [1]. However, it is difficult to differentiate clinically between bipolar and unipolar depressive episodes. According to Mitchell et al., patients with bipolar depression present more frequently symptoms of hypersomnia, hyperphagia, and psychomotor retardation [7]. However, this clinical approach is probabilistic and lacks specificity. Therefore, it is important to have clinical tools that permit an accurate differential diagnosis between unipolar and bipolar depressive episodes in order to improve treatment and prognosis.

Studies have shown that olfactory perception may be impaired in depression [8]. Patients suffering from depression may have impairments in their olfactory acuity and reduced olfactory sensitivity [9,10] that may recover after symptomatic remission [9,11]. They may also exhibit reduced levels of odor identification [12] and an alteration in their rating of an odor’s pleasantness (hedonic rating) [13,14]. However, very few studies compared bipolar and unipolar episodes when evaluating the olfactory function in depression [15]. The olfactory function may be also altered in some neurological and metabolic disorders. Olfactory memory is affected in Alzheimer’s disease [16]. Lietzau et al. showed that type 2 diabetes may impair olfactory memory and odor detection and decreases neurogenesis in the olfactory bulb [17]. Several hypotheses have been made to try to explain the olfactory dysfunction in diabetes, including the possibility that endocrine and vascular changes may lead to neuropathy and damage to the olfactory nerve [18,19]. Obesity and overweight are also associated with olfactory dysfunction [20]. Olfactory impairment seen in obesity can be related to various metabolic and endocrine defects, such as hyperleptinemia, hyperglycemia, and insulin resistance. However, weight loss through bariatric surgery may reverse the olfactory decline [21].

Olfactory recognition memory may be also affected by depression. Indeed, olfactory memory is more associated with emotions than auditory and visual memories [16,22]. According to Koster et al., olfactory perception and memory are implicitly associated with the emotional and hedonic rating of the environment. Subjects associate well-known odors with feelings of safety and comfort and will react immediately to unexpected odors [23]. Testing olfactory memory requires a time interval between the learning and the odor recognition phases. In long-term memory tasks, this interval may last from few minutes to several years. Subjects will encode odors during the first session and will try to identify the learned “old” odors among other “new” odors or distractors during the second session [16,24].

Several studies have evaluated olfactory recognition memory in depression. Zucco and Bollini showed that deficits in odor memory recognition were correlated with depressive symptoms. However, this study tested short-term olfactory memory, since the two sessions of testing were separated by only few seconds [12]. Naudin et al. (2014) [16] showed that olfactory memory is impaired in MDD. Depressed elderly patients had less correct responses and more wrong responses than healthy controls for both familiar and unfamiliar odors [16]. As for patients suffering from bipolar depressive episodes, no studies have been performed so far to evaluate olfactory memory in this population. The present study is the first to evaluate olfactory memory in bipolar depressed and euthymic patients.

The primary objective of this study was to evaluate long-term olfactory memory recognition in patients with unipolar and bipolar depression and to compare their olfactory memory function with those of patients in remission and healthy controls. The secondary objectives were the followings:

- To determine patients’ judgments of different parameters of olfaction (pleasantness, intensity, familiarity, and emotional aspect).
- To determine differences in olfactory memory recognition and judgments ratings between familiar and unfamiliar odors.
- To study the correlations between these olfactory judgments and clinical variables (severity of depression, onset and duration of illness, anhedonia, anxiety).

## 2. Methods

### 2.1. Participants

Patients were recruited in the inpatient and outpatient psychiatric units of two hospital settings (Psychiatric Hospital of the Cross and Hôtel-Dieu de France, Lebanon). The patients were divided into 4 clinical groups: Depressed Unipolar (DU), Depressed Bipolar (DB), Euthymic Unipolar (EU), and Euthymic Bipolar (EB). The control participants were recruited among healthy individuals with no history of any mood or psychotic disorder or any psychiatric treatment. The groups were matched based on key demographic characteristics such as age, sex, smoking status, and education level. Inclusion criteria for patients were the following: age between 18 and 64 years, current or past diagnosis of a depressive episode, absence of smell impairment related to any brain or nasal surgery or lesion, absence of current pregnancy, absence of current or past substance abuse disorders, in remission for more than 3 months for patients included in the euthymic groups. Exclusion criteria for all participants (patients and controls) were the following: presence of psychotic symptoms associated with depression, presence of (hypo)manic or mixed episodes, severe cognitive impairment, treatment with medication affecting olfaction, inability to undergo the assessment, and anosmia.

**Figure 1:**
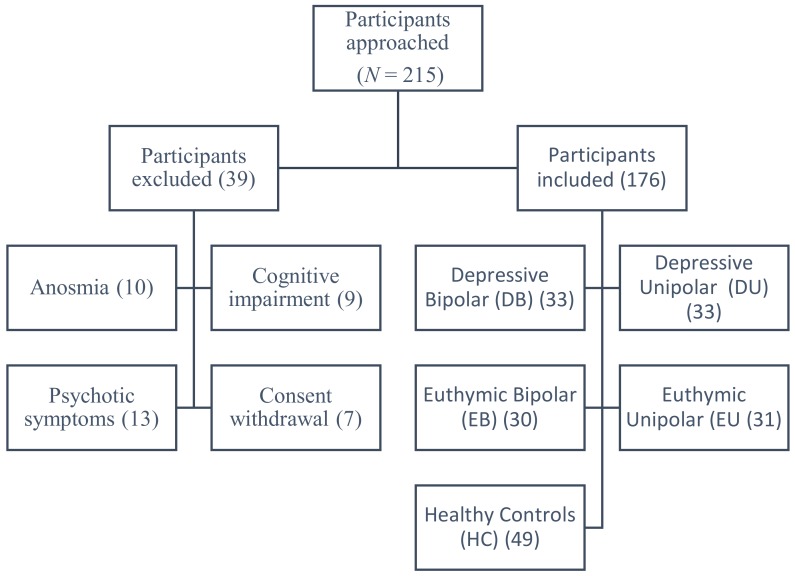
Flow diagram of participants’ recruitment.

Over 18 months, 215 participants were approached (Figure 1), and 176 participants were included in five groups: DU (*n* = 33), DB (*n* = 33), EU (*n* = 31), EB (*n* = 30), and healthy controls (HC; *n* = 49). Thirty-nine participants were excluded for the following reasons: anosmia (*n* = 10), cognitive impairment (*n* = 9), presence of psychotic symptoms (*n* = 13), and consent withdrawal (*n* = 7).

The study was approved by the local ethical committee board (Faculty of Medicine, Saint-Joseph University, Beirut, Lebanon) (Ethical code number: CEHDF 665 on 22/12/14) and conducted in accordance with Good Clinical Practice procedures and the current revision of the Declaration of Helsinki. All participants signed an informed consent. The participants did not receive any financial compensation for their participation in this study. The two evaluators in this study were a clinical psychiatrist and a clinical psychologist, both trained to use the scales and tests needed for this study.

### 2.2. Clinical Assessment

All participants had a 90–120 min interview to assess their clinical status and olfactory function. Interviewers obtained information concerning social and demographic status (age, marital status, educational level), present and past medical history, family psychiatric history, current and past treatments, number of depressive episodes, number of manic and hypomanic episodes, total duration of the depressive episodes, number of hospital admissions, age of onset of mood disorder and smoking status.

Clinical assessment included the following tools: the Mini International Neuropsychiatric Interview (MINI 5.0.0) [25,26] was used for the diagnosis of current and past psychiatric disorders; the Montgomery–Åsberg Depression Rating Scale (MADRS) [27] was used to assess the severity of depressive symptoms; the Young Mania Rating Scale (YMRS) [28] was used to confirm the absence of any manic, hypomanic, or mixed episode; the State–Trait Anxiety Inventory (STAI) [29] was used to evaluate the intensity of anxiety symptoms; the Chapman physical and social anhedonia questionnaire [30,31] was used to evaluate clinical anhedonia.

### 2.3. Olfactory Assessment

The assessment of olfactory memory was performed according to the procedure described by Naudin et al. [16]. At the beginning of the procedure, the participants were informed that they would undergo an olfactory test without specifying that it was a memory test. In order to evaluate long-term olfactory recognition memory, a yes–no recognition test was developed and included two sessions of olfactory stimuli. In the first session, a set of olfactory stimuli was presented, followed by a second set of stimuli one hour later, which comprised mixed odors of the first set along with novel odors [32,33]. In this protocol, the participants were asked to smell 8 odors in the first session and 16 odors in the second sessions (8 odors of the first sessions and 8 novel odors). After the first session, the participants were asked to rate the pleasantness (hedonic aspect), familiarity level, intensity, and emotional level of the perceived odors on a 10 cm linear scale labeled at each end (highly unpleasant/highly pleasant; unfamiliar odor/very familiar odor; low intensity/very intense; negative emotion/positive emotion). The resulting answer was expressed with a score ranging from 0 to 10.

**Figure 2:**
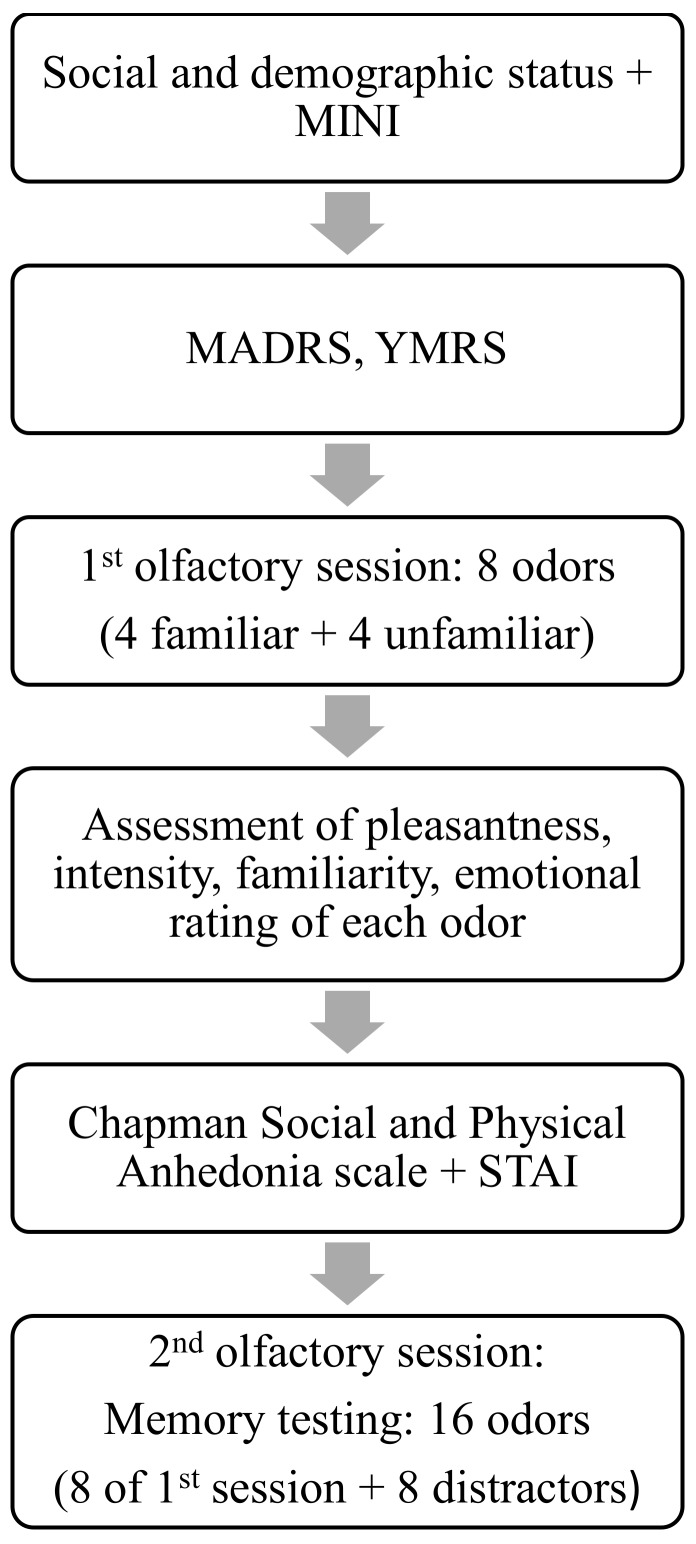
Consecutive steps of the assessment process. MADRS: Montgomery–Åsberg Depression Rating Scale; MINI: Mini-International Neuropsychiatric Interview; STAI: State–Trait Anxiety Inventory; YMRS: Young Mania Rating Scale.

This first session of the memory test allowed the participants to encode the odors. Among the 8 odors presented for encoding, 4 were unfamiliar (cetone V, pyralone, pandanol, and allyl amyl glycolate) and 4 were considered familiar, since regularly encountered in daily life (caramel, lavender, banana, and coconut). All odors were provided free of charge by Givaudan (Argenteuil, France). The odors’ concentrations were of equal intensity in order to avoid any influence of intensity on the memory process. Supra-threshold concentrations were used to ensure that all odors were well perceived by the participants [16]. In the second session, 16 odors were presented to the participants: the 8 odors from the first session and 8 news odors considered as “distractors”. The 8 distractors also consisted of 4 familiar odors (almond, coffee, jasmine, and orange) and 4 unfamiliar odors (aldehyde C11, caryophyllene, irival, and folrosia). For each of the 16 odors, the participants were asked whether they had smelled the specific odor during the first session or not. Thus, the participants indicated whether the stimuli in the second session had been previously presented (old odor) or not (new odor). Four categories of answers were then defined: (1) Hits: correct recognition (response of “old” for an old odor), (2) Misses: incorrect recognition (response of “new” for an old odor), (3) Correct rejection (response of “new” for a new odor), and (4) False alarms: false recognition (response of “old” for a new odor). Odors were presented in jars that prevented ingestion or spilling, in the same order for all participants. Each jar had a 3-digit random identification number. The time allowed to smell the odors was not limited. A 30 sec interval between odors was respected to prevent olfactory adaptation. The different steps in the assessment process are schematized in Figure 2.

### 2.4. Statistical Analysis

**Figure 3:**
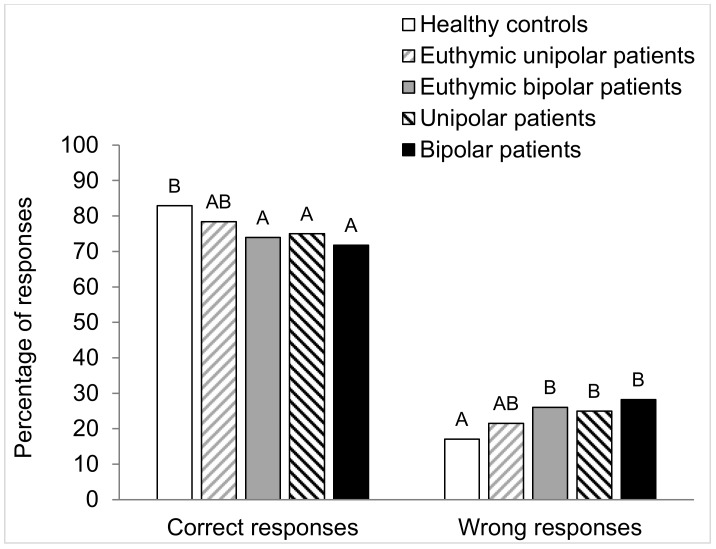
Between-groups comparison of the percentages of correct (correct rejections and hits) and wrong (misses and false alarms) responses. For each type of responses, values with the same letters are not significantly different at the 5% level of significance (Marascuilo procedure).

**Figure 4:**
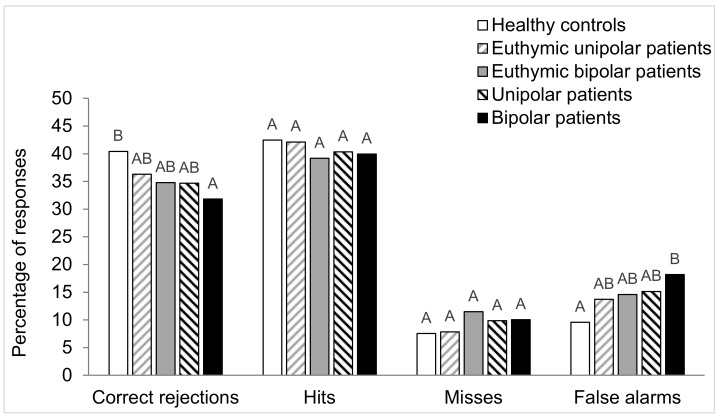
Between-groups comparison of the percentage of correct rejections, hits, misses, and false alarms responses. For each type of responses, values with the same letters are not significantly different at the 5% level of significance (Marascuilo procedure).

The results of the long-term odor recognition memory task were classified in the 4 categories of answers presented previously: 2 correct answers (hits and correct rejections) and 2 wrong answers (misses and false alarms). Figure 3 and Figure 4 present these types of responses expressed in percentage for each group of subjects. A detection index (DI) score was calculated, reflecting the subjects’ ability to discriminate between old and new odors [16].

**Figure 5:**
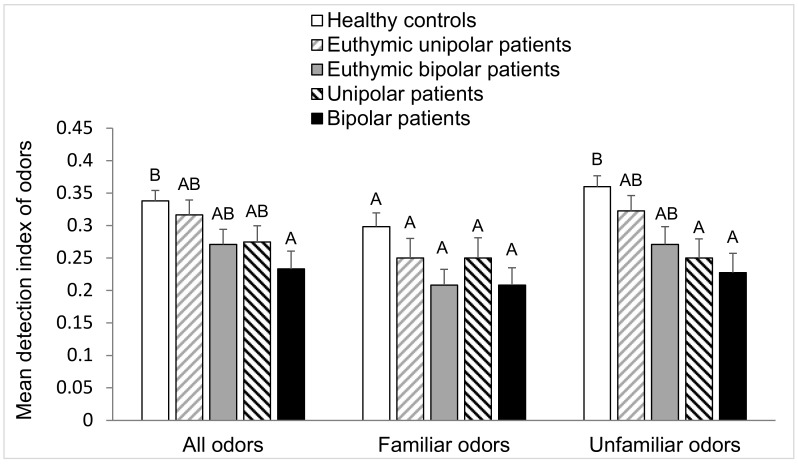
Between-groups comparison of mean detection indices of odors (all odors, only familiar odors, and only unfamiliar odors). For each group of odors (all, familiar, or unfamiliar), values with the same letters are not significantly different at the 5% level of significance (Tukey test). Vertical bars indicate the standard deviation of the mean.

The DI was calculated as follows:

DI = [p(hits) − p(false alarms)](1)

where p(hits) corresponds to the proportion of “yes” answers when the sample was an old odor, and p(false alarms) corresponds to the proportion of “yes” answers when the sample was a distractor (new odor). A high DI indicates that targets are clearly distinguished from distractors. Figure 5 presents the mean detection index of odors (with standard deviation of the mean) for each group of subjects.

**Figure 6:**
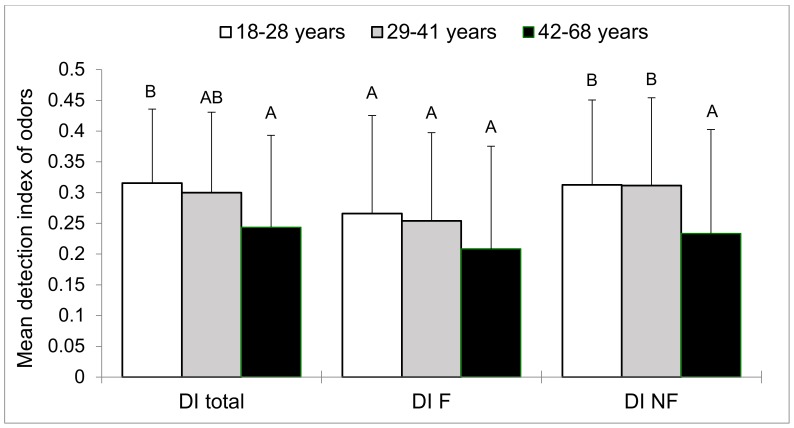
Between-age groups comparison of mean detection indices of odors (all odors: DI total; only familiar odors: DI F and only unfamiliar odors: DI NF). For each mean detection index of odors (all, familiar, or unfamiliar), values with the same letters are not significantly different at the 5% level of significance (Tukey test). Vertical bars indicate the standard deviation of the mean.

The Chi-square test was used to compare proportions of qualitative variables of the different groups of subjects (sex, smoking status, and number of correct and wrong responses). The Marascuilo procedure was performed for two-by-two comparisons of the different groups. The same statistical tests were also used to compare the number of each category of responses (hits, correct rejections, misses, and false alarms) for the five groups of subjects. The quantitative variables (age and educational level) of the five groups were computed separately with an analysis of variance (ANOVA) with one factor, i.e., group. As significant effect of group was found, and a two-by-two comparison between groups was carried out using the Tukey test. The Tukey test results are presented in the form of different letters (A, B, and C) in Table 1, Table 2 and Table 3 and in Figure 3, Figure 4, Figure 5 and Figure 6. Groups sharing the same letter are not significantly different, while groups who do not share the same letter have significant differences.

In order to control the effect of age on different variables (detection index score, pleasantness, familiarity, intensity, and emotion perception of the subjects), we divided the participants into three age categories: 1) 18–28 years (60 subjects), 2) 29–41 years (58 subjects), and 3) 42–68 years (58 subjects). This age criterion was made arbitrarily and created three equinumerous groups, which was crucial for statistical reasoning.

Two-way ANOVA (factors: group and age) was used to compare the DI scores between the five groups of subjects and in order to test the effect of age on the detection index score. Tukey multiple comparison tests were used for pairwise comparisons of groups. DI scores were evaluated for all odors and then for familiar and unfamiliar odors separately.

For each odor’s characteristic (pleasantness, familiarity, intensity, and emotion), analysis of variance with 3 factors and one interaction, i.e., stimulus (familiar and unfamiliar odors), group (5 groups of subjects: DU, DB, EU, EB, and HC), age (3 groups of subjects: 18–28 years, 29–41 years, and 42–68 years), and group × stimulus interaction, was carried out. As significant effects of age, group, and group × stimulus interaction were found, a two-by-two comparison between groups was carried out using the Tukey test.

The Pearson correlation coefficient was used to study the relationship between the clinical subjects’ state and their olfactory performances. The Pearson coefficient was calculated for the 4 patients’ groups and the significant results obtained in the different tests and scales.

All statistical analyses were performed at 95% confidence interval (alpha = 5%). They were conducted using XLSTAT-Pro software, version 2019.

## 3. Results

### 3.1. Demographic and Clinical Characteristics

To avoid factors of confusion affecting olfaction, the five groups of participants were matched according to age (F_(4,171)_ = 0.2, *p* = 0.95), sex (χ^2^ = 0.1; *df* = 4; *p* = 1), and smoking status (χ^2^ = 0.8; *df* = 4; *p* = 0.94). No significant difference between the groups was found also for educational level (F_(4,171)_ = 2.2, *p* = 0.07) (Table 1).

The mean number of (hypo)manic episodes was of 3.8 and 3.2 in the depressed and euthymic bipolar groups, respectively, while none of the controls or unipolar participants experienced any (hypo)manic episode. Concerning the patients, a significant group effect was highlighted for the number of depressive episodes (F_(3,123)_ = 7.1, *p* < 0.001) and for the number of hospital admissions (F_(3,123)_ = 8.9, *p* < 0.001). DB patients had significantly higher mean numbers of depressive episodes and hospital admissions (8.8 and 4.5, respectively) compared to EB (4.6 and 1.5), DU (3.5 and 1.7), and EU (2 and 0.1) patients. There was no difference between patients’ groups concerning the age of onset (F_(3,123)_ = 1, *p* = 0.4). As expected, a significant group effect was found for all clinical and psychometric parameters (MADRS: F_(4,171)_ = 507, *p* < 0.001; YMRS: F_(4,171)_ = 2.9, *p* = 0.02; physical anhedonia: F_(4,171)_ = 12, *p* < 0.001; social anhedonia: F_(4,171)_ = 12, *p* < 0.001; STAI-state: F_(4,171)_ = 60, *p* < 0.001; STAI-trait: F_(4,171)_ = 29, *p* < 0.001). The Tukey test showed that the scores on MADRS, anhedonia, and STAI scales were significantly higher in the depression groups (DB and DU) compared to both the euthymic groups (EB and EU) and the controls (HC) (Table 1).

### 3.2. Evaluation of Odors’ Pleasantness, Intensity, Familiarity, and Emotional Aspect

The three-way analysis of variance with interaction, indicated a significant effect of stimulus (pleasantness: F_(7,1366)_ = 155, *p* < 0.001; familiarity: F_(7,1366)_ = 96, *p* < 0.001, emotion: F_(7, 1366)_ = 110, *p* < 0.001), and group (pleasantness: F_(4,1366)_ = 18, *p* < 0.001; familiarity: F_(4,1366)_ = 16, *p* < 0.001, emotion: F_(4,1366)_ = 14, *p* = 0.001) for three olfactory parameters: pleasantness, familiarity, and emotion. A significant effect of age was found only for familiarity (F_(2,1366)_ = 3.8, *p* = 0.02) and intensity (F_(2, 1366)_ = 3.5, *p* = 0.03); (pleasantness: F_(2,1366)_ = 1.1, *p* = 0.32; emotion: F_(2,1366)_ = 1.7, *p* = 0.19). Concerning intensity, a significant effect was found for stimulus (F_(7,1366)_ = 11, *p <* 0.001), and no significant group effect was demonstrated (F_(4,1366)_ = 2, *p* = 0.1). No significant “group × stimulus” interaction was found for all olfactory parameters (pleasantness: F_(28,1366)_ = 1.3, *p* = 0.16; familiarity: F_(28,1366)_ = 1.2, *p* = 0.18, intensity: F_(28,1366)_ = 1.1, *p* = 0.27, emotion: F_(28,1366)_ = 0.9, *p* = 0.67).

Table 2 shows the mean ratings of different groups for the pleasant, familiar, intense, and emotional aspects of the eight odors presented during the first session. The Tukey test showed that DB and DU participants rated odors as significantly less familiar than EU and HC participants, thus showing that patients in depressed phases recognize odors as less familiar than healthy controls. As for pleasantness rating, DB participants rated the odors as significantly less pleasant than participants in other groups (DU, EB, EU, and HC). All groups had a significantly lower pleasantness rating compared to HC. DB had a lower emotional rating than EB. All groups had a significantly lower emotional rating compared to HC (Table 2).

Table 3 shows the mean ratings of the three different age groups for the pleasant, familiar, intense, and emotional aspects of the eight odors presented during the first session. The Tukey test showed that the 18–28 years group rated odors as significantly less familiar than the 29–41 years group. As for intensity rating, the 42–68 years participants rated odors as significantly less intense than the 18–28 years group. 

### 3.3. Long-Term Odor Recognition

#### 3.3.1. Correct and Wrong Responses

Concerning the total correct responses (hits and correct rejections), the Chi-squared test showed a significant difference between the five groups (χ^2^ = 27. 9; *p* < 0.001). The Marascuilo procedure demonstrated less correct answers in DB, DU, and EB patients compared to healthy controls (Figure 3). For the total wrong responses (misses and false alarms), the Chi-squared test demonstrated a significant difference between the five groups (χ^2^ = 27.9; *p* < 0.001). The Marascuilo procedure showed that the DU, DB, and EB groups committed more errors than the healthy controls (Figure 3).

#### 3.3.2. Hits, Correct Rejections, Misses, and False Alarms

Concerning the comparison of the five groups for each responses category separately, the Chi-squared test revealed a difference only for correct rejection (χ^2^ = 11.4; *p* = 0.022) and false alarms (χ^2^ = 21.4 *p* < 0.001) but not for hits (χ^2^ = 2.0; *p* = 0.74) and misses (χ^2^ = 7.4; *p* = 0.12). The Marascuilo procedure showed that DB participants exhibited more false alarms and less correct rejections than HC, indicating an alteration in the screening of new odors. No significant difference was found in the mean number of hits and misses between the five groups of participants (Figure 4).

#### 3.3.3. Detection Index

Considering all odors, the two-way analysis of variance, indicated a significant effect of age (F_(2,169)_ = 4.9, *p* = 0.009) and group (F_(4,169)_ = 3.6, *p* = 0.008) concerning the participants’ ability to distinguish targets (old odors) between distractors (new odors). The Tukey test showed that DB patients has a significantly lower detection index than HC (Figure 5). The Tukey test also showed that young subjects (18–28 years) has a significantly higher detection index than the 42–68 years participants (Figure 6). No difference in detection index was seen between groups regarding familiar odors (F_(4,169)_ = 2.3, *p* = 0.07). No significant effect of age was also demonstrated (F_(2,169)_ = 2.3, *p* = 0.1). For unfamiliar odors, a significant difference between the groups in the ability to distinguish between targets and distractors was revealed (F_(4,169)_ = 5.3, *p* < 0.001). The detection index was found to be lower for DB and DU patients than for healthy volunteers (Figure 5). A significant age effect was shown (F_(2,169)_ = 5.9, *p* = 0.003). The Tukey test highlighted that the 42–68 years group of subjects had a significantly lower detection index than the other two groups (Figure 6).

### 3.4. Correlations between Clinical and Olfactory Variables

Correlation coefficients were calculated for all patients’ groups between olfactory variables (DI and pleasantness ratings for all odors and for familiar and unfamiliar ones) and clinical variables (MADRS, STAI, anhedonia score, number of depressive episodes, number of hospital admissions, total duration of depressive episodes, age at first episode). In the DU group, the DI was negatively correlated with the MADRS score (r = −0.4; *p* = 0.02) and the number of admissions (r = −0.35; *p* = 0.046) and positively correlated with the pleasantness rating of familiar odors (r = 0.39; *p* = 0.025). In the DB group, a positive correlation was found between DI and the pleasantness rating of familiar odors (r = 0.4; *p* = 0.023). The DI of unfamiliar odors and the age at first episode were negatively correlated (r = −0.35; *p* = 0.045). In the EU group, a significant correlation was found between the pleasantness rating of familiar odors and DI (r = 0.47; *p* = 0.007) and the number of depressive episodes (r = −0.39; *p* = 0.029). The DI of unfamiliar odors was negatively correlated with the total duration of depressive episodes (r = −0.37; *p* = 0.039). In the EB group, the total duration of depressive episodes was significantly correlated with the pleasantness rating of familiar (r = −0.38; *p* = 0.041) and unfamiliar odors (r = −0.42; *p* = 0.020).

## 4. Discussion

This study evaluated the olfactory recognition memory of four groups of patients with unipolar and bipolar depression, in symptomatic and euthymic phases. We compared subjects’ results between different groups as well as with those of healthy controls in order to identify olfactory memory alterations that would persist after remission. Participants’ ratings of the presented odors allowed us to evaluate other variables that are the hedonic and emotional judgments of odors as well as their perceived intensity and familiarity. The aim of the study was to uncover olfactory memory deficits in depression that may constitute either state or trait markers of these disorders. We also searched for olfactory differences between unipolar and bipolar depression that may be considered as differentiation markers, with possible consequences for the therapeutic approach to these two forms of depression. Therefore, we present the only study that has evaluated so far olfactory recognition memory in bipolar depression.

Our results showed that olfactory recognition memory is altered in both unipolar and bipolar depression patients compared to healthy individuals. This alteration persists in euthymic bipolar patients after remission of the depressive episode. Olfactory memory deficits also increased with the severity of depression, as shown by the negative correlation between odor detection index and MADRS scores and number of hospital admissions in unipolar depressed subjects. The orbitofrontal cortex (OFC) is involved in the cognitive impairments seen in depression as well as in odor memory and the hedonic value given to olfactory stimuli [34,35,36], which explains the olfactory memory deficits observed in our study. However, whilst the deficit in olfactory recognition memory improves after remission of unipolar episodes, patients with bipolar disorders will maintain their deficits in euthymic states. These results suggest that odor recognition memory may be a potential state marker of unipolar depressive episodes, while being a trait marker of bipolarity.

The deficit in olfactory memory was more pronounced in bipolar depressed patients regarding the screening of new odors (higher number of false alarms and lower number of correct rejections), suggesting that bipolar depression may be a more severe form of depression affecting the cognitive capacity of patients to screen and process new stimuli. The amygdala and the hippocampus are both parts of the limbic system that are involved in the processing of memory and emotions and in the judgment of olfactory stimuli [37,38,39,40]. Studies have found dysfunctions in amygdala and hippocampal activities in depression [41,42] that may explain also the olfactory memory deficits observed in our study. Deficits in olfactory recognition in depression do not only regard new stimuli but also unfamiliar ones. Indeed, our results show that subjects in depressed phases (unipolar and bipolar) have lower detection of unfamiliar odors compared to controls, which may indicate the presence of a cognitive bias in favor of well-known and encoded odors in depression. Moreover, these results were confirmed in the judgment rating tasks were depressed subjects (unipolar and bipolar) rated odors as less familiar than controls and euthymic unipolar subjects. As for euthymic bipolar subjects, they also had a lower familiarity rating than controls. Furthermore, our results show a significant correlation only between odor detection index and hedonic rating of familiar odors. Therefore, olfactory memory impairment in depression may be due to cognitive processing errors, whereby known odors will be considered as less familiar thus affecting the emotional value attributed to stimuli and exacerbating memory deficits. This lack of familiarity recognition is more pronounced in bipolar patients and persists after remission. This may constitute a possible state marker for depression and a trait marker for bipolarity.

As for the judgment rating of different odors, this study shows that hedonic (pleasantness) and emotional ratings were both affected in depressed individuals. Subjects with depression (DU and DB) and euthymic phases (EU and EB) attributed lower hedonic and emotional values to the tested odors compared to healthy controls, suggesting that these ratings may constitute trait markers of unipolar and bipolar depression. The hedonic rating of depressed bipolar patients was the lowest compared to all the other groups and improved significantly after remission (EB group), without reaching the ratings of healthy controls, thus characterizing the state of bipolar depression. Hedonic rating was also associated with the duration of bipolar depression, as shown in the negative correlation between hedonic rating of odors and the cumulative duration of depressive episodes seen in the EB group. Studies have indeed found that depression is associated with a lower hedonic rating of olfactory stimuli [9,10,13]. The hedonic judgment is processed in the OFC and modulated by emotional states. It also depends on the processing of the prefrontal cortex (PFC) [38]. Depression can be associated with dysfunctions in these regions that may lead to the observed deficits in pleasantness rating [15]. Furthermore, the difference seen in hedonic rating between DU and DB subjects may help differentiating between unipolar and bipolar depressive episodes and may be considered a differentiation marker of bipolar depression. 

Our study showed differences in olfaction across different ages categories. Older subjects tended to rate odors with lower intensity than young adults and had a lower olfactory detection index compared to younger subjects. Several studies reported a reduction of olfactory function with age [43,44]. More specifically, olfactory memory deficits increase in older adults [45]. Olfactory deficits associated with aging can be attributed to several etiologies, including structural and functional abnormalities of the olfactory system as well as changes in neurotransmitter processes [44,46]. Therefore, our results, showing increased olfactory deficits in older adults, are in concordance with the literature.

To summarize, this study shows significant differences between subjects with unipolar and bipolar depressive disorders and controls concerning olfactory recognition memory and hedonic and emotional rating of odors. Different brain regions are indeed involved in the affective states seen in unipolar and bipolar depression and in the processing of olfactory stimuli. The PFC and the OFC are implicated, as well as other regions of the limbic system (amygdala, hippocampus). Dysfunctions of these different brain regions may affect the olfactory perception in bipolar and depressive disorders [8,15].

This is the first study that evaluated the olfactory recognition memory in subjects with bipolar disorder and compared it with those of subjects with depressive disorder and healthy controls. We showed that depressed patients exhibited lower levels of olfactory memory. Deficits in olfactory memory were more pronounced in patients with bipolar disorder and affected more new and unfamiliar odors. The patients also exhibited alterations in the pleasantness and emotional rating of odors. These differences in olfactory perception may constitute potential state and trait markers for depressive and bipolar disorders. They also provide strong evidence that sensory olfactory markers may be useful clinical tools in differentiating between unipolar and bipolar depressive episodes.

Some limitations of this study have to be acknowledged. First, this study excluded subjects with severe cognitive impairment, since they were unable to complete the assessment. This may have affected the results by excluding severely depressed patients. Second, this study may have a recall bias, since patients’ history was taken directly from the subjects and not from systematized medical files. Third, this study did not evaluate the effect of medication on olfaction. Most included subjects (except healthy controls) had psychotropic treatment. Despite the fact that taking a medication with any effect on olfaction was an exclusion criterion of this study, this limitation has to be acknowledged. Fourth, this is a cross-sectional study comparing different groups of subjects in symptomatic depression and euthymic phases. A study comparing the same subjects in depressive and euthymic phases would have given more accurate results. To minimize this bias, we matched participants’ groups for age, sex, and smoking status. As stated earlier, the olfactory function may be altered by obesity or diabetes. Depressed patients may be treated by medications that increase the risk of obesity and the likelihood of diabetes. However, these two aspects were not controlled in our groups, thus constituting a limitation of our study. Finally, the judgment of olfactory stimuli may be different between populations, cultures, and countries. The results of this study reflect the odor ratings of the Lebanese population, which may be different from those of populations in other countries.

## 5. Conclusions

In conclusion, this study showed that olfactory memory may constitute a possible marker of depression and bipolarity. Alterations in olfactory perception may also be helpful in understanding the physiopathology of mood disorders. However, their use in daily clinical practice is still unavailable. So far, clinicians only rely on clinical scales/questionnaires and patients’ history to differentiate between unipolar and bipolar depression. However, these scales/questionnaires can be biased, may vary across cultures, and need validation for different populations. Developing olfactory testing for depression may provide more objective measures of diagnosis than simple clinical scales, even if such tests will need standardization and validation across cultures/populations. Indeed, the replication of these results and the development of standardized practical olfactory tests for clinical use may open the gate for new diagnostic tools in depressive and bipolar disorders. Moreover, the olfactory stimulation of depressed patients should also be considered as a possible therapeutic tool based on sensory emotional stimulation.

## Figures and Tables

**Table 1:** Demographic and clinical characteristics of the participants.

Patients’ Groups	HC (*n* = 49)	DB (*n* = 33)	EB (*n* = 30)	DU (*n* = 33)	EU (*n* = 31)
Mean age, SD	35 (12.1)	36.6 (10.3)	36.2 (13.4)	36.8 (9.9)	34.9 (14.0)
Female/male, ratio	38/11	25/8	23/7	25/8	24/7
Smokers/non-smokers, ratio	21/28	16/17	15/15	17/16	14/17
Educational level, mean (SD) *	3.0 (0.2)	2.1 (0.7)	3.5 (5.2)	2.1 (0.7)	2.7 (0.6)
Marital status, %					
- Single	63.3	54.5	60	45.5	74.2
- Married	36.7	36.4	23.3	45.5	22.6
- Divorced	0	9.1	10	3	3.2
- Widowed	0	0	6.7	6	0
Age of onset, mean (SD)	-	24.5 (8.7)	25.1 (11.3)	28.3 (9.6)	24.9 (10.9)
Depressive episodes, n	0	8.8 (10.9) ^B^	4.6 (4.2) ^A^	3.5 (3.7) ^A^	2 (1.5) ^A^
(Hypo-)manic episodes, n	0	3.8 (5.1)	3.2 (4.0)	0	0
Hospital admissions, n	0	4.5 (6.1) ^B^	1.5 (2.7) ^A^	1.7 (1.9) ^A^	0.1 (0.4) ^A^
Total duration of depression (months), mean (SD)	0 (0)	36.7 (64.7)	24.6 (29.7)	23.6 (33.3)	15.3 (15.1)
Use of psychotropic treatment (%)	0	93.9	83.3	90.1	48.4
MINI 5.0.0 (%)					
- MDE, current episode	0	100	0	100	0
- MDE, lifetime	0	100	100	100	100
- Suicidal risk, last month	0	75.8	0	75.8	0
- (Hypo-)mania, lifetime	0	100	100	0	0
- Panic disorder, lifetime	2	15.2	13.3	12.1	12.9
- Agoraphobia, current episode	8.2	36.4	3.3	12.1	12.9
- Social phobia, current	4.1	24.2	10	30.3	9.7
- GAD, last 6 months	4.1	48.5	20	60.6	6.5
- OCD, last month	0	3	0	6.1	0
- PTSD, last month	0	3	0	9.1	0
- Alcohol abuse, last 12 months	0	0	0	6.1	0
- Cannabis abuse, last 12 months	0	0	3.3	0	0
- Psychotic disorder, lifetime	0	0	0	0	0
- Eating disorders, last 3 months	0	0	0	0	3.2
MADRS, mean (SD)	1.6 (2.9) ^A^	41.3 (8.3) ^B^	2.0 (2.0) ^A^	39.3 (8.4) ^B^	2.2 (2.2) ^A^
YMRS, mean (SD)	0.1 (0.5) ^A^	0.6 (1.6) ^A^	0.8 (1.4) ^A^	0.5 (0.9) ^A^	0.1 (0.4) ^A^
Physical anhedonia, mean (SD)	14.0 (7.1) ^A^	24.0 (8.7) ^B^	16.4 (9.5) ^A^	25.0 (9.0) ^B^	17.0 (9.0) ^A^
Social anhedonia, mean (SD)	9.9 (5.7) ^A^	18.8 (6.2) ^C^	14.4 (7.8) ^B^	19.6 (6.0) ^C^	13.0 (5.6) ^AB^
STAI-trait, mean (SD)	39.8 (9.5) ^A^	60.7 (9.3) ^B^	44.3 (10.6) ^A^	55.4 (11.1) ^B^	44.3 (8.6) ^A^
STAI-state, mean (SD)	31.4 (9.0) ^A^	61.1 (14.3) ^B^	31.3 (9.0) ^A^	57.8 (15.6) ^B^	31.7 (9.3) ^A^

* Educational level was calculated on a three-level scale (1, 2, and 3, corresponding to primary, secondary, and university levels, respectively. DB: depressed bipolar; EB: euthymic bipolar; DU: depressed unipolar; EU: euthymic unipolar; GAD: Generalized Anxiety Disorder; HC: healthy controls; MDE: Major Depressive Episode; MINI 5.0.0: Mini-International Neuropsychiatric Interview version; PTSD: Posttraumatic Stress Disorder; OCD: Obsessive Compulsive Disorder; SD: Standard Deviation. For each clinical and psychometric parameter, if the means share the same letter, they are not significantly different at the 5% level of significance (Tukey test).

**Table 2:** Mean scores (standard deviation) for the olfactory judgments of all odors evaluated by HC, EU, EB, DU, and DB patients. For each olfactory judgment, if the means share the same letter, they are not significantly different at the 5% level of significance (Tukey test).

Olfactory Judgment	HC (*n* = 49)	EU (*n* = 31)	EB (*n* = 30)	DU (*n* = 33)	DB (*n* = 33)
**Pleasantness, mean (SD)**	4.7 (0.1) ^C^	4.1 (0.2) ^B^	3.9 (0.2) ^B^	4.2 (0.2) ^B^	3.0 (0.2) ^A^
**Familiarity, mean (SD)**	5.7 (0.2) ^C^	5.3 (0.2) ^BC^	4.5 (0.2) ^AB^	4.3 (0.2) ^A^	4.0 (0.2) ^A^
**Intensity, mean (SD)**	6.93 (0.1)	7.3 (0.2)	7.2 (0.2)	6.8 (0.2)	6.8 (0.2)
**Emotion, mean (SD)**	5.0 (0.1) ^C^	4.3 (0.2) ^B^	4.2 (0.2) ^B^	4.1 (0.2) ^AB^	3.5 (0.2) ^A^

**Table 3:** Mean scores (standard deviation) for the olfactory judgments of all odors evaluated by the 18–28 years group, the 29–41 years group, and the 42–68 years group. For each olfactory judgment, if the means share the same letter, they are not significantly different at the 5% level of significance (Tukey test).

Olfactory Judgment	18–28 Years Group (*n* = 60)	29–41 Years Group (*n* = 58)	42–68 Years Group (*n* = 58)
**Pleasantness, mean (SD)**	3.9 (3.5)	3.9 (3.6)	4.1 (3.4)
**Familiarity, mean (SD)**	5.0 (3.8) ^B^	4.5 (3.7) ^A^	4.8 (3.7) ^AB^
**Intensity, mean (SD)**	7.2 (2.4) ^B^	7.0 (2.7) ^AB^	6.8 (2.7) ^A^
**Emotion, mean (SD)**	4.0 (3.3)	4.2 (3.4)	4.4 (3.3)
